# Optimizing soil health through activated acacia biochar under varying irrigation regimes and cultivars for sustainable wheat cultivation

**DOI:** 10.7717/peerj.18748

**Published:** 2025-01-17

**Authors:** Lubaba Komal, Summera Jahan, Atif Kamran, Abeer Hashem, Graciela Dolores Avila-Quezada, Elsayed Fathi Abd_Allah

**Affiliations:** 1 Institute of Botany, University of the Punjab, Lahore, Pakistan; 2 Botany and Microbiology Department, College of Science, King Saud University, Riyadh, Saudi Arabia; 3 Facultad de Ciencias Agrotechnologicas, Universidad Autonoma de chihuahua, Chihuaha, Mexico; 4 Plant Production Department, College of Food and Agricultural Sciences, King Saud University, Riyadh, Saudi Arabia

**Keywords:** Activated biochar, Water scarcity, Antioxidants, Soil microporosity, Wheat yield, Photosynthetic pigments, Organic matter

## Abstract

Wheat, a staple food crop globally, faces the challenges of limited water resources and sustainable soil management practices. The pivotal elements of the current study include the integration of activated acacia biochar (AAB) in wheat cultivation under varying irrigation regimes (IR). A field trial was conducted in the Botanical Garden, University of the Punjab, Lahore during 2023–2024, designed as a split-split-plot arrangement with RCBD comprising three AAB levels (0T, 5T, and 10T, T = tons per hectare) three wheat cultivars (Dilkash-2020, Akbar-2019, and FSD-08) receiving five IR levels (100%, 80%, 70%, 60%, and 50% field capacity). Biochar amended soil showed improved BET surface area, pore size, and volume. Carbon recovery (45%) and carbon sequestration capacity (49%) of 10T-AAB amended soil were better than non-amended soil (0.43% and 0.13%, respectively). The 10T-AAB amendment significantly improved the soil’s microporosity and water retention capacity, increasing it by 1.1 and 2.2 times, respectively. Statistical analysis showed that a reduction in IR negatively affected plant growth and yield. The 10T-AAB levels significantly increased sugar contents (14%), relative water content (10–28%), membrane stability index (27–55%), and photosynthetic pigments (18–26%) of wheat leaves under deficit irrigation among all the cultivars. Maximum stress markers (catalase, proline, peroxidase, and superoxide dismutase) were observed from Akbar under 50% irrigation with 0T-AAB, and the least were observed from 50% irrigated Dilkash-2020 with 10T-AAB amended soil. Among cultivars, Dilkash-2020 was observed to be the best for maximum yield, followed by FSD-08 and Akbar-2019, respectively. When compared to other IR levels, 10T-AAB amended soil had the highest yield enhancement (12, 11, and 9.2 times for Dilkash-2020, FSD-08, and Akbar-2019, respectively). Hence, AAB enhanced wheat production by improving soil properties, drought resilience, and yield attributes.

## Introduction

Water scarcity, driven by climate change, is altering evapotranspiration patterns, soil moisture level, and plant rhizosphere dynamics (Albacete, Martínez-Andújar & Pérez-Alfocea, 2014). Addressing these challenges requires targeted specific crop management strategies that support water availability during stress intervals while enhancing crop productivity (El Chami et al., 2019). Such strategies can boost crop water use efficiency, reduce surface runoff, and limit deep percolation losses (Capraro et al., 2018). In regions where fertile soils and water resources are limited, improving wheat production (a globally crucial crop) is essential for food security (Huang et al., 2022). Wheat faces ongoing challenges from both water scarcity and the need for sustainable soil management practices (Yu et al., 2020).

Biochar has gained attention as an amendment that enhances soil’s physicochemical properties and moisture retention (Shakeel et al., 2022; Jahan et al., 2022). Biochar is produced through pyrolysis of organic substances at a very high temperature; it offers soil a high surface area and micropore volume, crucial for moisture retention and water conservation (Lehmann & Joseph, 2015). Activation of biochar, particularly organic methods like vermicompost or perlite addition, is reported to enhance these benefits further (Sanchez-Hernandez, Ro & Díaz, 2019). The combination of biochar with perlite and vermicompost supports improved water retention through a combined effect of hydrated volcanic glass and decomposing organic matter, fostering a more resilient rhizosphere (Jahan et al., 2022).

Activated biochar contributes to soil structure, water retention, and favorable plant morpho-physiological and biochemical responses (Lehmann & Joseph, 2015). It aids in soil health recovery, enhanced water use efficiency, and sustained plant growth, even under water-limited conditions (Iqbal et al., 2024). Additionally, biochar’s log-term carbon retention enhances carbon sequestration, promoting sustainable soil health and productivity (Daer et al., 2024). Such improvements in soil conditions support plant water uptake, reduce reactive oxygen species (ROS) production, and stabilize photosynthetic pigments and sugar contents under stress (Wu et al., 2023). Consequently, biochar helps mitigate water-deficit stress impacts by increasing antioxidant enzyme activity, such as peroxidases and superoxide dismutase (Shakeel et al., 2022). Various field studies are increasingly documenting the positive effects of biochar; however, plant responses significantly vary depending on the type of biochar, activation methods, and biomass properties (Jahan et al., 2024).

Despite its proven benefits, field studies on biochar application, especially activated biochar, are limited in the context of deficit irrigation for wheat production. Exploring its integration with precision irrigation could lead to enhanced crop resilience and productivity. This study fills the knowledge gap by investigating activated biochar’s role in supporting wheat under deficit irrigation, contributing to soil health, and sustaining agricultural productivity. Given these conditions, we hypothesize that soil amended with activated biochar will support wheat growth and productivity under deficit irrigation conditions by improving soil moisture retention and enhancing soil physicochemical properties. The primary objective of this study is to assess the synergistic effects of activated biochar on soil quality, plant physiology, growth, and yield in three commercial wheat cultivars (Dilkash-2020, Akbar-2019, and FSD-08) under variable irrigation regimes. This study will help to identify the optimal biochar amendment and irrigation regime combination for water-scarce areas, promoting sustainable agriculture and food security. This research is novel in that it examines organically activated biochar tailored to wheat production under water-stressed conditions and evaluates their potential in practical field settings. The findings aim to bridge critical gaps in sustainable crop management by developing our understanding of activated biochar’s role in improving water stress resilience in wheat.

## Materials and Methods

### Production of activated biochar

Wood twigs of *Acacia nilotica* were utilized for biochar production as optimized by Jahan et al. (2022). Before pyrolysis, raw biomass was air-dried to reduce its moisture content. Production of biochar was carried out by the slow pyrolysis technique at 450 °C for a three-hour duration using a batch pyrolysis temperature-controlled unit. After the cooling, the physico-chemical properties of biochar were analyzed by Jahan et al. (2023). For activation purposes, biochar, vermicompost, and perlite were mixed in a 1:1:1 ratio along with molasses to speed up the process and incubated for thirty days. Mixing and turning of the material was done daily to maintain proper aeration. After incubation, samples of the activated acacia biochar were assessed to determine its physicochemical characteristics (Jahan et al., 2023).

### Experimental design and area

A field trial was executed at Botanical Garden, University of the Punjab, Lahore, Pakistan (N31°30′4.3236″, E74°18′5.4684), during 2023–2024. The experiment comprised of split-split plot arrangement with a randomized complete block design (RCBD) in triplicate with a plot size of 5.95 m^2^. Factors under observation comprised of activated acacia biochar (0T-AAB, 5T-AAB, and 10T-AAB), cultivars (Dilkash-2020, Akbar-2019, and FSD-08), and irrigation regimes (100%, 80%, 70%, 60%, and 50% field capacity). Activated acacia biochar (AAB) was applied manually to the topsoil (15 cm) and thoroughly mixed as 2.7 and 5.4 kg per plot for 5 and 10 tons per hectare, respectively. Cultivars were selected as per recommended cultivars for irrigated soils from the Ayub Agricultural Research Institute (AARI), Faisalabad. Basal fertilizer doses for nitrogen, phosphorous, and potassium were applied in the form of urea (20.4 g per plot), SOP (36 g per plot), and DAP (54 g per plot), but urea was applied in two splits with a second dose in subsequent irrigation.

### Meteorological data

**Figure 1:**
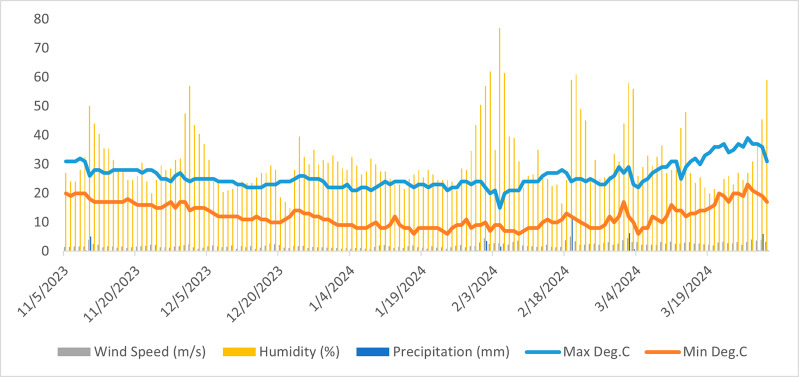
Crop meteorological data retrieved from crop monitoring system database presenting variation in Max Deg.C (maximum temperature in °C), Min Deg.C (minimum temperature in °C), wind speed (m/s), specific humidity (m/s), humidity (%), and precipitation (mm).

Meteorological data was obtained from EOS data analytics: Crop Monitoring System (https://crop-monitoring.eos.com/). Parameters of meteorological data specifically included, minimum and maximum temperature (°C), wind speed (m/s), specific humidity (%), and precipitation (mm) (Fig. 1).

### Strategy for maintaining irrigation regimes

Water requirements of crop was calculated by the Eq. (1) presented by the Food and Agriculture Organization (FAO).

(1) \begin{eqnarray*}\mathrm{IN}=\mathrm{ETc}-\mathrm{Pe}\end{eqnarray*}

where IN presents the net water requirement, ETc stands for evapotranspiration of crop, and Pe shows effective rainfall. Moreover, evapotranspiration was estimated by using the expression (Eq. 2) as given by Mehta & Pandey (2015),

(2) \begin{eqnarray*}ETc=ETo\times Kc\end{eqnarray*}

where Eto is reference evapotranspiration and Kc is crop coefficient. Reference evapotranspiration was determined by using Penman Monteith Eq. (3) Mehta & Pandey (2015),

(3) \begin{eqnarray*}E{T}_{o}= \frac{0.14\Delta \left( Rn-G \right) +\gamma \left[ \frac{900}{T+273} \right] {U}_{2}({e}_{s}-{e}_{a})}{\Delta +\gamma (1+0.34{U}_{2})} \end{eqnarray*}

where T is the daily mean temperature (°C) at a height of 2 m, Rn indicates net radiation, G presents the soil heat flux in MJm^2^/day, Δ presents the gradient of the vapor pressure-temperature curve in KPa/°C, *γ* is the psychometric constant (KPa/°C), U_2_ shows the wind rate per day at a 2 meter elevation in meters per second, and e_s_ and e_a_ present the average and real saturation vapor pressure, respectively. Reference evapotranspiration (ETo) was calculated by CROPWAT 8.0 (Soomro et al., 2023). The effective rainfall was observed as given below (Eq. 4).

(4) \begin{eqnarray*}Pe=(0.6\times \mathrm{P})-3.33.\end{eqnarray*}

If P ≤ 70 mm, Pe and P show effective rainfall and total precipitation respectively. Irrigation regimes (80% to 50%) were calculated using the percentage formula, based on evapotranspiration patterns. Soil moisture content was observed using a moisture meter (Lutron PMS-714) at regular intervals before each irrigation to estimate the field capacity.

## Soil and biochar physicochemical analysis

Soil pH and electrical conductivity were estimated using a pH and EC meter by following the standard procedure of Rayment & Lyons (2011). Standard procedures by Estefan, Sommer & Ryan (2013) were followed for estimation of water holding capacity, soil porosity, and pore size. The yield of activated biochar was assessed using Eq. (5). Brunauer-Emmett-Teller (BET) and Barrett-Joyner-Halenda (BJH) were used for soil particle surface area analysis, pore size and volume analysis using Quantachrome Instruments version 11.04 with nitrogen gas media. Carbon recovery (CR) was estimated using Eq. (6) (Li et al., 2022). Mean residence time (MRT) and carbon (%) remaining in soil over 100 years (HC_+100_), were calculated according to Eqs. (7) and (8), respectively (Venkatesh et al., 2022). H/C shows the atomic ratio of activated biochar and amended soils. The letter ‘e’ represents an exponential term. As shown in Eq. (9), R50 presents an indicator of carbon’s recalcitrance in amended soil and activated biochar (Harvey et al., 2012; Li et al., 2022), whereas, T50_Graphite_ and T50_Biochar_ are temperatures required for 50% weight loss of activated graphite and biochar, respectively. Graphite was used as reference substance with purity ≥ 99.85% and 100 mesh. Equation (10) was used to estimate the carbon sequestration potential of activated biochar (Venkatesh et al., 2022).

(5) \begin{eqnarray*}Yield= \frac{Biochar~weight}{Raw~weight} \times 100\end{eqnarray*}

(6) \begin{eqnarray*}Carbon~Recovery \left( CR \right) = \frac{{C}_{Biochar}}{{C}_{Biomass}} \times Yield\end{eqnarray*}

(7) \begin{eqnarray*}MRT=4501\times {e}^{-3.2\times \frac{H}{C} }\end{eqnarray*}

(8) \begin{eqnarray*}H{C}_{+100}=1.05-0.616\times \frac{H}{C} \end{eqnarray*}

(9) \begin{eqnarray*}{R}_{50}= \frac{{T}_{50~Biochar}}{{T}_{50~Graphite}} \end{eqnarray*}

(10) \begin{eqnarray*}Carbon~sequestration={R}_{50}\times CR.\end{eqnarray*}

### Plant physiological and biochemical analysis

Wheat leaves were analyzed for leaf proline content at the grain filling stage by following Bates, Waldren & Teare (1973). The method of DuBois et al. (1956) was used to evaluate the sugar contents in the leaf sample. Lipid peroxidation in the leaf sample was analyzed by the method of Procházková, Boušová & Wilhelmová (2011) where malondialdehyde (MDA) was the indicator of lipid peroxidation. The membrane stability index (MSI) of the leaf was assessed by method given by Sairam (1994). The method of Mullan & Pietragalla (2012) was followed for relative water content (RWC). The Arnon method was used to find the chlorophyll content (Arnon, 1949) while carotenoid content was assessed by the method of Lichtenthaler & Wellburn (1983). Protein content was estimated by the method of Bradford (1976). Beauchamp & Fridovich (1971) method was used to observe the activity of superoxide dismutase (SOD). The peroxidase (POD) level was analyzed following the method of Gorin & Heidema (1976) and the method of Iwase et al. (2013) was used to analyze the catalase activity in the leaf sample.

### Plant growth and yield analysis

Plant growth and yield parameters were analyzed at the grain filling stage and a digital analytical balance (Model FA2204E, China) was used to measure the fresh and dry weights. The method of Usman, Liedl & Shahid (2014) was used to determine apparent water productivity as follows:

(11) \begin{eqnarray*}\text{Apparent}~\text{water}~\text{productivity}~ \left( \mathrm{Kg}~{\mathrm{m}}^{-3} \right) = \frac{Seed~Yield~(kg~h{a}^{-1})}{Irrigation~Water~({m}^{-3})} .\end{eqnarray*}

### Statistical analysis

Experimental data was analyzed using IBM SPSS Statistics 23.0 software for analysis of variance (ANOVA) and post-hoc comparisons, including Duncan’s test for alphabetic arrangement of data ranges. Descriptive statistics were used for generating graphs based on means and standard deviation. Pearson correlation was generated through Origin 2024 software (https://www.originlab.com/2024). The PCA analysis and heatmap were constructed to predict the correlation of treatments with growth variables of wheat grown under varying irrigation regimes using RStudio (R-4.3.1-x86 64.pkg) (R Core Team, 2023; RStudio Team, 2023).

## Results

### Soil and biochar physicochemical analysis

Table 1 presents the physicochemical soil analysis. The organic matter (OM) was highest in soil with 10T-AAB amendment, reaching 5.2%. There was 1.25 fold higher organic carbon in soils amended with 10T-AAB as compared to 0T-AAB amendment. The highest percent carbon value was observed by 10T amended soil with 3% carbon, whereas nitrogen content peaked in 5T-AAB amendment with 1.07%. Soil water holding capacity (WHC) was at its maximum in 10T-AAB amended soil with 28%, which is 37% higher than non-amended soil. Macropore space was highest at 118% under 0T-AAB amendment. Porosity was highest in 10T-AAB amended soil, at 269.38 (1.1 folds higher). The pH levels remained stable, peaking at 6.7, *i.e.,* near to neutral pH and effective for wheat growth in 10T-AAB amended soil. The 10T-AAB amended soil showed slightly increased electrical conductivity (EC). Other attributes, including hydrogen, oxygen, carbon recovery, carbon sequestration capacity, and mean residence time, were increased in 10T-AAB amended soil (Table 1).

**Table 1:** Physicochemical properties of activated acacia biochar (AAB), non-amended soil (0TAAB AS), 5 tons per hectare (5T-AAB AS) and 10 tons per hectare (10T-AAB AS) amended soil.

**Traits**	**AAB**	**0T-AAB AS**	**5T-AAB AS**	**10T-AAB AS**
C	63.78^a^	2.31^d^	2.61^c^	2.90^b^
H	3.20^a^	0.25^c^	0.28^b^	0.31^b^
O	12.04^d^	19.47^a^	19.34^b^	17.10^c^
N	0.76^d^	0.83^c^	1.07^a^	1.04^b^
H/C	0.60^b^	1.35^a^	0.42^c^	0.35^d^
O/C	0.14^d^	6.33^a^	5.56^b^	4.42^c^
Ash	13.50^a^	9.65^d^	10.08^c^	10.26^b^
Volatile Matter	33.70^a^	3.26^d^	5.83^c^	7.27^b^
Organic matter (%)	53.30^a^	4.15^d^	4.69^c^	5.22^b^
WHC (%)	92.73^a^	20.43^d^	24.03^c^	27.93^b^
Pore Space (%)	98.29^d^	117.58^a^	115.58^b^	110.58^c^
Porosity (%)	429.75^a^	244.98^d^	254.29^c^	269.37^b^
BET SA (m2 g-1)	8.390^c^	4.360^d^	20.730^a^	19.136^b^
BJH PS (Å)	15.158^c^	19.732^a^	15.753^b^	15.078^d^
BJH PV (cc g-1)	0.003^c^	0.003^c^	0.007^a^	0.006^b^
Ph	6.92^a^	5.30^d^	6.10^c^	6.70^b^
EC	1.27^a^	0.64^d^	0.76^c^	0.84^b^
Yield (%)	53.36^a^	8.95^d^	39.24^c^	46.48^b^
CR (%)	70.56^a^	0.43^d^	34.10^c^	44.97^b^
CS (%)	25.61^c^	0.13^d^	36.46^b^	48.78^a^
MRT (y)	663.45^c^	59.45^d^	1172.18^b^	1478.29^a^
HC+100 (%)	68.04^c^	21.84^d^	79.13^b^	83.44^a^

**Notes.**

C = Carbon, H = Hydrogen, O = Oxygen, N = Nitrogen, H/C= Hydrogen to carbon ratio, O/C= Oxygen to carbon ratio, EC= Electrical conductivity, SA= surface area, PS = Pore size, PV= Pore volume, CR = Carbon recovery, CS = Carbon Sequestration, MRT = Mean residence time, HC_+100_ = the percent of the carbon that would remain in the soil after 100 years and the unit of MRT in the table is year (y), Data presenting Mean and various superscripted alphabets indicate statistical significance at 95% confidence interval. AAB= activated acacia biochar, 0T-AAB AS= non amended soil, 5T-AAB AS = 5 tons per hectare activated acacia biochar amended soil, and 10T-AAB AS = 10 tons per hectare activated acacia biochar amended soil.

**Figure 2:**
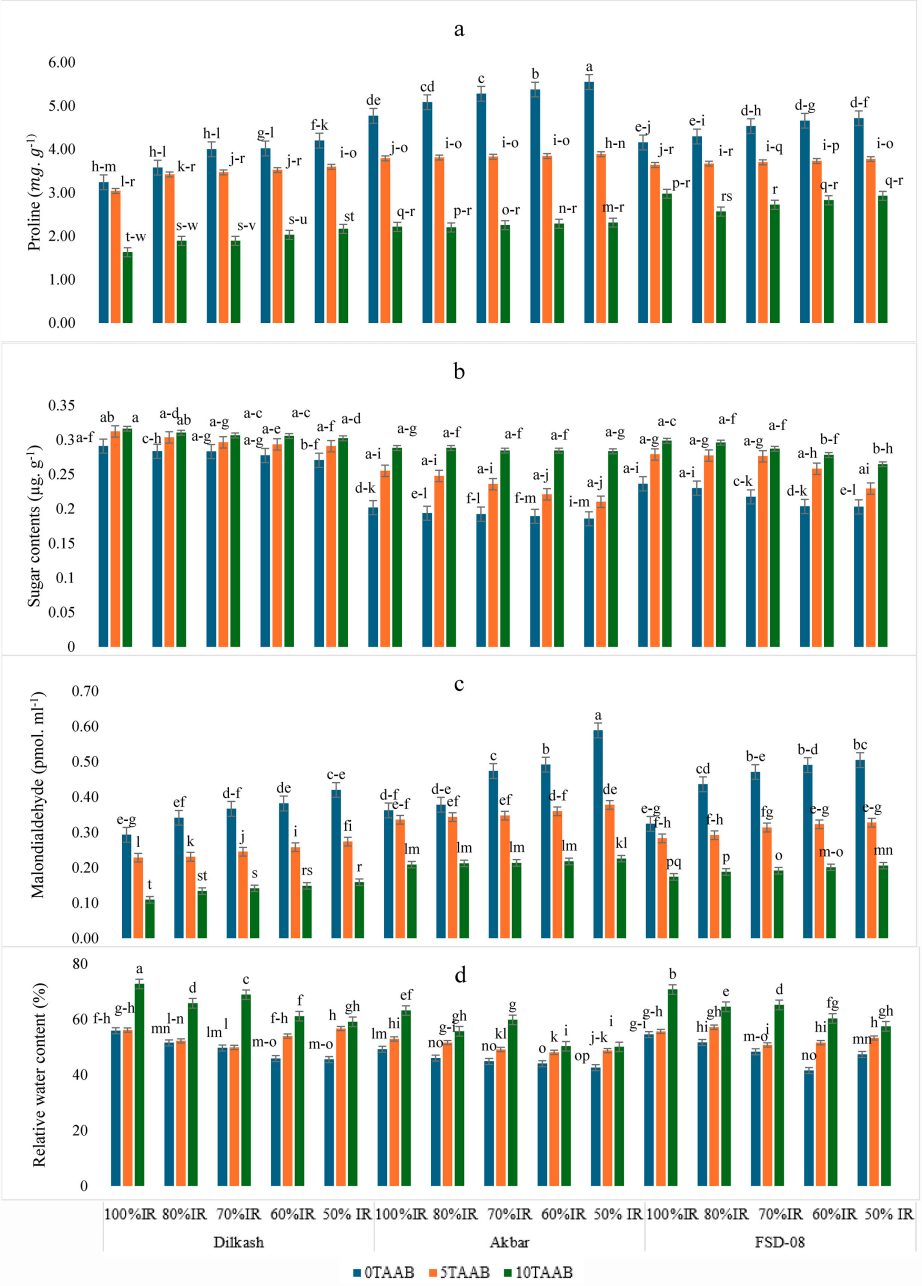
Mean comparison for the effect of varying irrigation regimes, AAB and cultivars. (A) Proline (mg g^−1^), (B) sugar contents (mg g^−1^), (C) malondialdehyde (pmol ml^−1^), and (D) relative water content. Lowercase letters indicate statistical significance at 95% confidence interval, 0T, 0 tons per hectare; 5T, 5 tons per hectare; 10T, 10 tons per hectare; AAB, activated Acacia Biochar; and IR, irrigation regime; Dilkash, Dilkash-2020 cultivar; Akbar, Akbar-2019 cultivar; and FSD-08, FSD-08 cultivar.

### Plant physiological and biochemical attributes

The analysis of variance showed a significant effect of activated acacia biochar (AAB) on the proline content of wheat cultivars under varying irrigation regimes. The mean comparison showed that 10T-AAB reduced proline content by 48%, 58%, and 39% in Dilkash-2020, Akbar-2019, and FSD-08, respectively, in 50% IR when compared to 0T-AAB (Fig. 2A). Sugar contents were reduced with a reduction in IR by 7%, 8%, and 14% in Dilkash-2020, Akbar-2019, and FSD-08, respectively, in 50% IR as compared to 100% irrigated plants in 0T-AAB (Fig. 2B). The AAB significantly reduced the MDA content under deficit IR (Fig. 2C). Results showed that AAB amendment significantly improved RWC, MSI, and other physiological attributes of wheat under varying irrigation regimes. Compared to control (0T-AAB), 5T-AAB and 10T-AAB increased RWC by 10% and 28%, respectively, in Dilkash-2020 (Fig. 2D).

**Figure 3:**
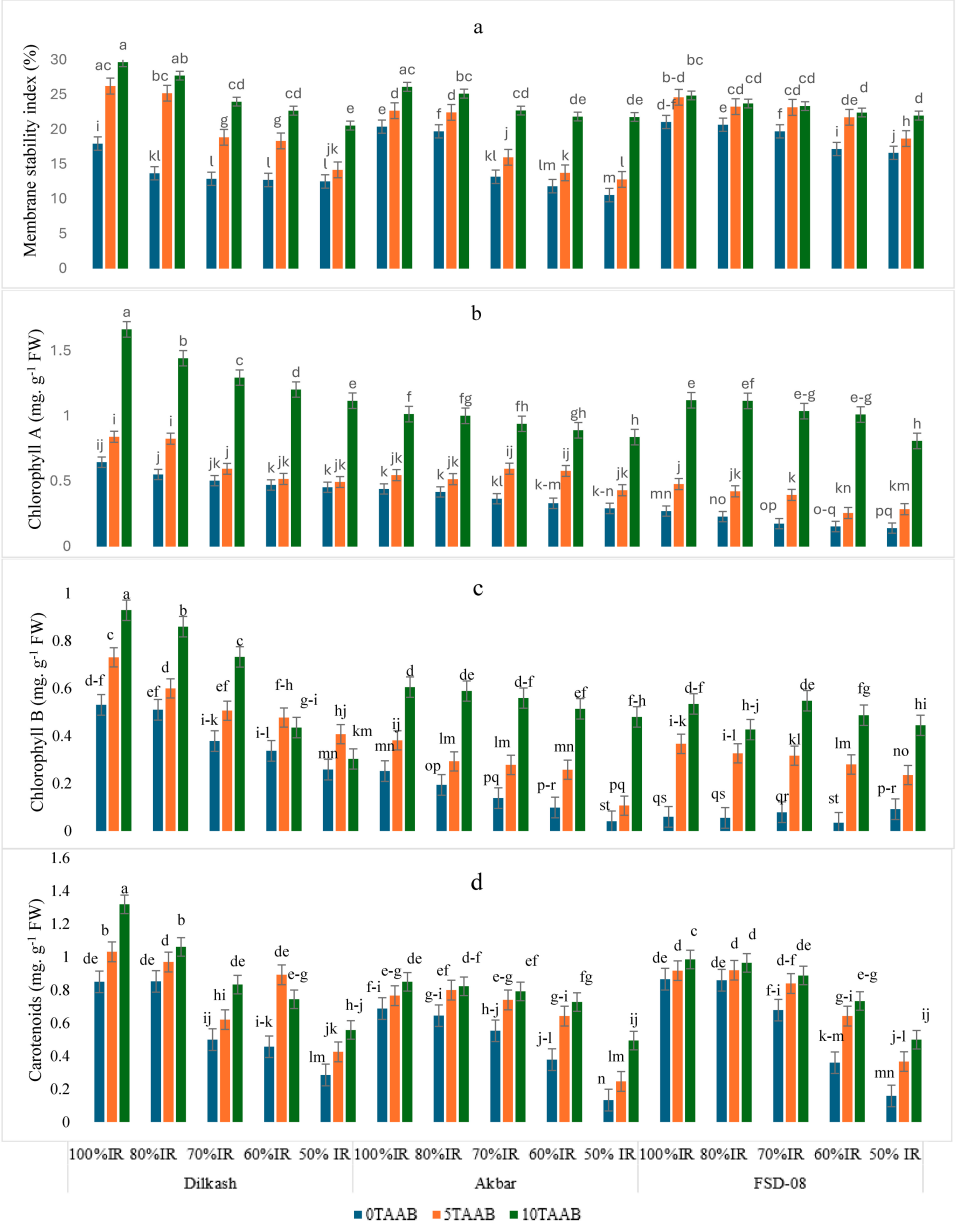
Mean comparison for the effect of varying irrigation regimes, AAB and cultivars. (A) Membrane stability Index (%), (B) chlorophyll a (mg g^−1^ FW), (C) chlorophyll b (mg g^−1^ FW), and (D) carotenoids (mg g^−1^ FW) in fresh leaves of wheat at grain filling stage. Lowercase letters indicate statistical significance at 95% confidence interval, 0T, 0 tons per hectare; 5T, 5 tons per hectare; 10T, 10 tons per hectare; AAB, activated Acacia Biochar; and IR, Irrigation regime; Dilkash, Dilkash-2020 cultivar; Akbar, Akbar-2019 cultivar; and FSD-08, FSD-08 cultivar.

Deficit IR reduced MSI by 20–50% with 0T-AAB. Whereas 5T-AAB and 10T-AAB improved overall MSI by 27% and 55%, respectively (Fig. 3A). The significant effect of biochar on photosynthetic pigments were observed under deficit IR. Compared to control (0T-AAB), 5T-AAB and 10T-AAB increased Chl a content by 18 and 26 times, respectively (Fig. 3B). whereas 5T-AAB and 10T-AAB produced Chl b content 18 and 27 times higher, respectively (Fig. 3C) and maximum carotenoids content was observed from 10T-AAB with 100% IR in Dilkash-2020 (Fig. 3D).

**Figure 4:**
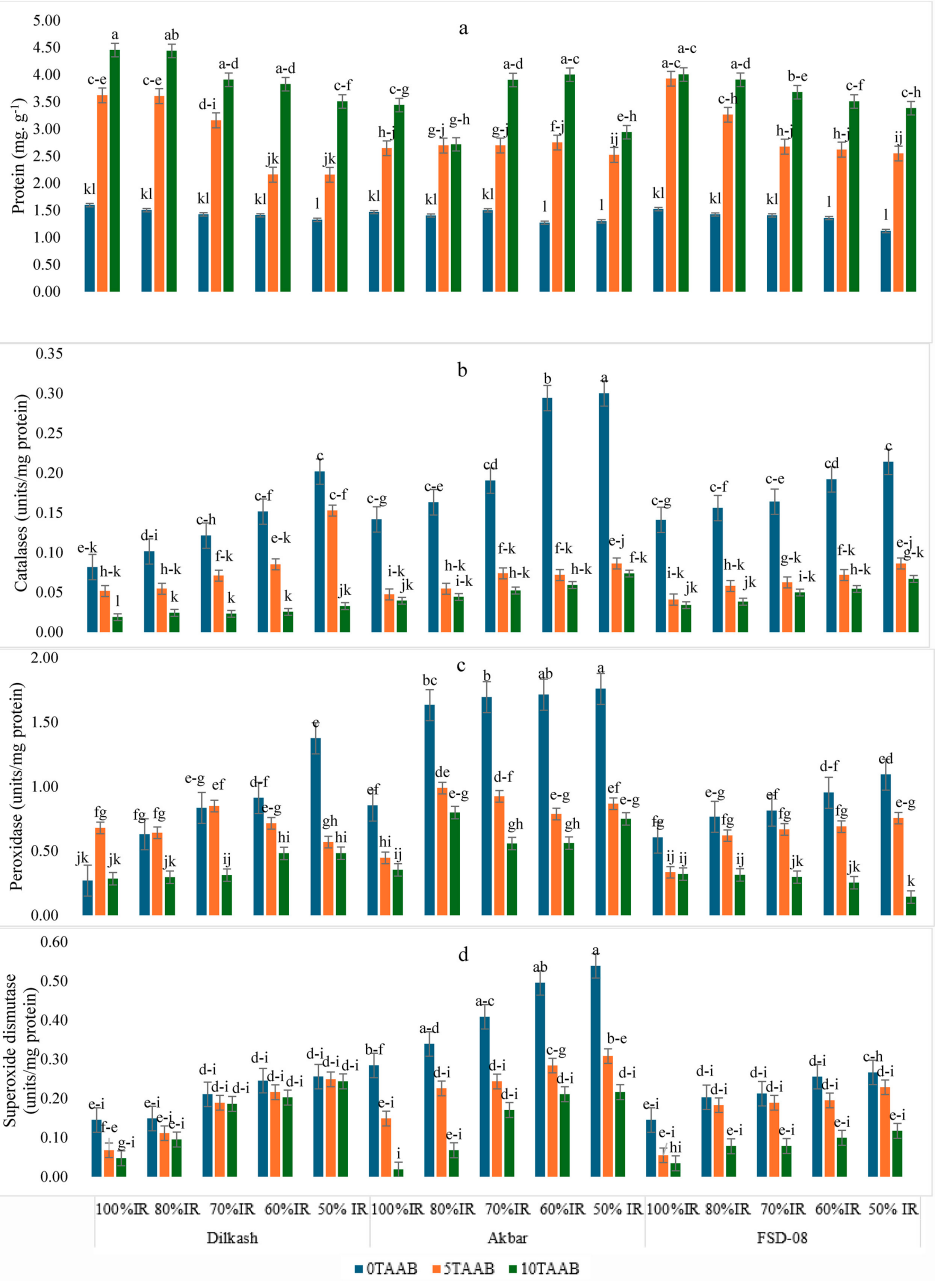
Mean comparison for the effect of varying irrigation regimes, AAB and cultivars. (A) Protein (mg g^−1^) (B) catalases (units/mg protein), (C) peroxidases (units/mg protein), and (D) superoxide dismutase (units/mg protein) in fresh leaves of wheat at grain filling stage; lowercase letters indicate statistical significance at 95% confidence interval 0T, 0 tons per hectare; 5T, 5 tons per hectare; 10T, 10 tons per hectare; AAB, activated Acacia Biochar; and IR, Irrigation regime; Dilkash, Dilkash-2020 cultivar; Akbar, Akbar-2019 cultivar; and FSD-08, FSD-08 cultivar.

Biochar amendment in low IR was observed to increase the protein contents in all cultivars but the major increase (18 times higher) was observed in Dilkash-2020 with 10T-AAB in 70% IR as compared to its counterpart with 0T-AAB followed by 50% IR with 10T-AAB (Fig. 4A). For catalase (Fig. 4B) and peroxidase (Fig. 4C), the peak level was in Akbar-2019 at 50% IR with 0T-AAB. Superoxide dismutase had the highest value in Akbar-2019 at 50% IR as well (Fig. 4D). The AAB amendment in deficit irrigation reduced the antioxidant activity by decreasing these enzymes levels by 17–57% in all cultivars.

### Plant growth and yield attributes

**Figure 5:**
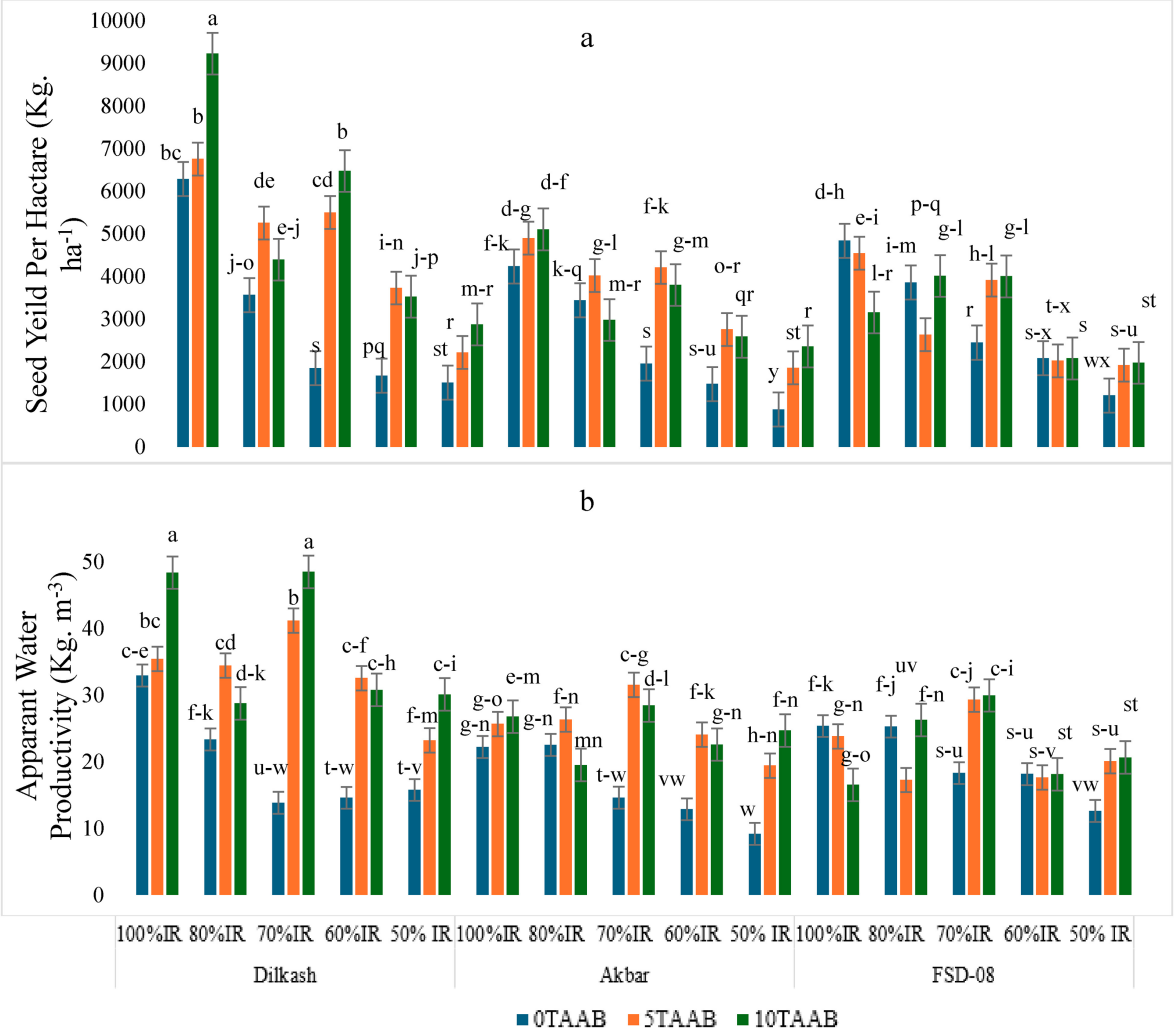
Mean comparison for the effect of varying irrigation regimes, AAB and cultivars on (A) seed yield per hectare (kg ha^−1^) and (B) apparent water productivity (kg m^−3^) of wheat. Where alphabets indicate statistical significance at 95% confidence interval, 0T, 0 tons per hectare; 5T, 5 tons per hectare; 10T, 10 tons per hectare; AAB, activated Acacia biochar; and IR, Irrigation regime; Dilkash, Dilkash-2020 cultivar; Akbar, Akbar-2019 cultivar; and FSD-08, FSD-08 cultivar.

There was a significant (*p* ≤ 0.05) effect of AAB on plant growth indices and irrigation regimes significantly affected plant growth except for the number of tillers and leaves. The difference between cultivars for the number of tillers and root length was not significant. Plant morphological traits including leaf fresh weight, stem fresh weight, root fresh weight, leaf dry weight, stem dry weight, and root dry weight showed significant increases with biochar (5T-AAB and 10T-AAB), and percent increases ranged from 16% to 81%, respectively, compared to the control (0T-AAB) under deficit IR conditions (Tables 2 & 3). It was observed that reduction in IR significantly decreased plant yield by 77%, 81%, and 82% in Dilkash-2020, FSD-08, and Akbar-2019, respectively (Table 4). But when these cultivars were grown in amendment with AAB, increased yield attributes were observed with both 5T-AAB (114%, 112%, and 88%, respectively) and 10T-AAB (119%, 110%, and 92%, respectively). The highest yield was observed from 100% IR with 10T-AAB in Dilkash-2020 and Akbar-2019 cultivars, but 5T-AAB in 70% IR gave the best grain yield for FSD-08. For spike length, spike weight, number of spikes per plant, spikelet per spike, and grains per spike, 10T-AAB in 70% IR proved to be the best (Table 4). Maximum yield per hectare and highest apparent water productivity were observed from Dilkash-2020 with 10T-AAB in 100%, followed by 70% IR (Figs. 5A and 5B).

**Table 2:** Means comparison for the effect of varying irrigation regimes, AAB levels of amendment and cultivars on leaf fresh weight (g), stem fresh weight (g), root fresh weight (g), leaf dry weight (g), stem dry weight (g) of wheat.

		**Leaf fresh weight (g)**			**Stem fresh weight (g)**			**Root fresh weight (g)**			**Leaf dry weight (g)**			**Stem dry weight (g)**		
**Cult.**	**IR**	**0T-AAB**	**5T-AAB**	**10T-AAB**	**0T-AAB**	**5T-AAB**	**10T-AAB**	**0T-AAB**	**5T-AAB**	**10T-AAB**	**0T-AAB**	**5T-AAB**	**10T-AAB**	**0T-AAB**	**5T-AAB**	**10T-AAB**
**Dilkash-2020**	**100%IR**	5.13 ± 0.15^c^	6.95 ± 1.27^ef^	5.39 ± 0.2^b^	16.30 ± 0.70^ef^	22.93 ± 0.09^b^	22.96 ± 1.03^b^	4.84 ± 0.60^c^	4.92 ± 0.40^bc^	6.19 ± 0.24^a^	0.95 ± 0.01^a^	1.84 ± 0.06^a^	4.06 ± 0.70^a^	11.31 ± 1.6^ef^	10.86 ± 1.4 ^abc^	10.51 ± 1.6 ^abc^
	**80%IR**	4.53 ± 0.35^cd^	3.28 ± 0.04^fg^	4.47 ± 0.1^cd^	29.37 ± 0.80^a^	16.47 ± 0.20^de^	19.03 ± 1.40^c^	4.72 ± 1.02^c^	3.24 ± 0.40^de^	5.30 ± 0.04^b^	0.92 ± 0.00^ef^	1.72 ± 0.06^b^	2.96 ± 0.02^b^	6.58 ± 0.2^d^	7.91 ± 0.8^de^	7.90 ± 0.5^d^
	**70%IR**	3.47 ± 0.34^f^	5.19 ± 0.3^b^	7.11 ± 1.2^a^	16.61 ± 0.20^ef^	28.16 ± 0.30^a^	25.72 ± 0.90^a^	3.45 ± 0.40^d^	4.18 ± 1.60^cd^	4.70 ± 1.06^bc^	0.88 ± 0.02^abc^	1.64 ± 0.00^bc^	2.83 ± 0.04^bc^	5.55 ± 0.2^ef^	6.15 ± 0.2^fg^	6.59 ± 0.2^e^
	**60%IR**	2.97 ± 0.3^gh^	3.63 ± 0.42^ef^	5.01 ± 0.1^b^	14.75 ± 0.60^fg^	13.98 ± 0.90^fg^	15.07 ± 1.10^de^	2.43 ± 0.09^ef^	1.93 ± 0.21^fg^	3.40 ± 0.30^de^	0.85 ± 0.02^bc^	1.63 ± 0.00^bc^	2.73 ± 0.04^cd^	5.09 ± 0.08^fgh^	5.38 ± 0.1^gh^	6.09 ± 0.3^ef^
	**50% IR**	2.05 ± 0.29^ij^	4.49 ± 0.38^de^	3.87 ± 0.05^e^	12.24 ± 0.40^h^	14.13 ± 0.40^fg^	14.86 ± 0.90^de^	0.92 ± 0.11^h^	1.80 ± 0.40^fg^	3.00 ± 0.04^e^	0.83 ± 0.02^cd^	1.56 ± 0.00^cd^	2.66 ± 0.02^d^	3.11 ± 0.9^j^	4.66 ± 0.3^hi^	5.19 ± 0.1^gh^
**Akbar-2019**	**100%IR**	5.15 ± 1.6^c^	4.07 ± 0.7^de^	4.04 ± 1.1^de^	22.67 ± 0.80^c^	15.03 ± 0.08^ef^	16.39 ± 0.50^d^	7.32 ± 2.00^a^	5.23 ± 0.40^b^	5.30 ± 0.40^b^	0.41 ± 0.04^ef^	1.23 ± 0.00^e^	2.10 ± 0.03^f^	15.08 ± 4.1^a^	14.98 ± 3.6^a^	10.84 ± 6.3^abc^
	**80%IR**	2.51 ± 0.1^hi^	3.98 ± 0.01^ef^	3.84 ± 1.14^efg^	9.48 ± 0.4^i^	12.95 ± 3.25^gh^	14.35 ± 0.90^e^	5.73 ± 0.15^b^	3.46 ± 0.34^de^	4.57 ± 0.48^bc^	0.27 ± 0.04^gh^	1.21 ± 0.02^e^	1.99 ± 0.02^g^	8.74 ± 0.3^c^	10.66 ± 0.9^bc^	11.10 ± 0.8^ef^
	**70%IR**	2.16 ± 0.14^hi^	2.91 ± 0.08^h^	3.43 ± 0.4^f^	9.11 ± 0.60^i^	10.90 ± 1.30^hi^	11.77 ± 0.30^fg^	1.64 ± 0.34^fg^	2.65 ± 0.20^ef^	3.58 ± 0.44^de^	0.23 ± 0.01^hi^	1.08 ± 0.07^f^	1.96 ± 0.00^gh^	7.73 ± 0.1^cd^	8.72 ± 0.5^cd^	9.32 ± 0.8^cd^
	**60%IR**	1.72 ± 0.2^jk^	1.84 ± 0.17^j^	3.12 ± 0.8^g^	12.74 ± 0.90^h^	8.29 ± 0.99^j^	11.87 ± 0.90^fg^	1.52 ± 0.47^fg^	3.27 ± 1.08^de^	3.52 ± 0.30^de^	0.22 ± 0.01^i^	1.02 ± 0.00^fg^	1.94 ± 0.00^gh^	7.13 ± 0.1^d^	7.68 ± 0.1^de^	8.66 ± 0.2^d^
	**50% IR**	1.44 ± 0.1^k^	1.34 ± 0.08^jk^	4.48 ± 0.3^cd^	8.40 ± 0.90^j^	10.51 ± 0.05^hi^	13.66 ± 2.75^ef^	0.55 ± 0.08^h^	2.54 ± 0.12^ef^	3.48 ± 0.50^de^	0.13 ± 0.01^j^	0.97 ± 0.00^g^	1.86 ± 0.01^h^	5.97 ± 1.2^def^	5.81 ± 1.4^fg^	6.25 ± 0.5^ef^
**FSD-08**	**100%IR**	6.13 ± 0.84^ef^	3.49 ± 1.1^f^	4.39 ± 0.3^cd^	12.08 ± 0.50^h^	17.99 ± 0.50^cd^	18.99 ± 0.01^c^	3.50 ± 0.24^d^	4.69 ± 0.28^bc^	3.99 ± 0.90^cd^	0.73 ± 0.01^de^	1.34 ± 0.32^d^	2.58 ± 0.02^e^	11.34 ± 2.3^ef^	8.77 ± 1.5^cd^	9.15 ± 1.1^cd^
	**80%IR**	3.65 ± 0.88^cd^	2.17 ± 0.10^i^	3.28 ± 0.01^fg^	12.65 ± 0.60^h^	12.93 ± 0.30^gh^	15.43 ± 0.07^de^	2.41 ± 0.32^ef^	4.01 ± 0.44^cd^	3.69 ± 0.49^de^	0.60 ± 0.01^fg^	1.48 ± 0.00^c^	2.49 ± 0.03^e^	6.44 ± 0.2^de^	6.65 ± 0.8^ef^	7.28 ± 0.6^de^
	**70%IR**	2.05 ± 0.24^i^	1.74 ± 0.38^j^	3.17 ± 0.2^g^	15.58 ± 0.70^f^	12.42 ± 0.20^gh^	13.71 ± 0.20^ef^	2.01 ± 0.37^f^	4.03 ± 0.71^cd^	4.09 ± 0.60^cd^	0.65 ± 0.00^ef^	1.43 ± 0.00^c^	2.38 ± 0.04^ef^	5.79 ± 0.2^ef^	5.17 ± 0.4^hi^	6.11 ± 0.2^ef^
	**60%IR**	1.52 ± 0.2^jk^	1.51 ± 0.2^jk^	2.70 ± 0.2^h^	9.56 ± 0.70^i^	10.77 ± 0.05^hi^	11.87 ± 3.01^fg^	1.52 ± 0.47^fg^	3.27 ± 1.08^de^	4.49 ± 0.60^bc^	0.54 ± 0.00^f^	1.40 ± 0.02^d^	2.21 ± 0.05^f^	4.28 ± 0.1^hi^	4.59 ± 0.1^hi^	5.57 ± 0.2^fg^
	**50% IR**	1.26 ± 0.2^k^	1.89 ± 0.4^j^	2.68 ± 0.5^hi^	7.53 ± 0.20^j^	10.51 ± 0.60^hi^	12.55 ± 0.05^fg^	0.92 ± 0.11^h^	2.53 ± 0.56^ef^	2.81 ± 0.05^e^	0.48 ± 0.04^fg^	1.36 ± 0.02^d^	2.14 ± 0.00^fg^	3.36 ± 0.5^ij^	4.07 ± 0.3^i^	4.73 ± 0.5^h^

**Notes.**

Data presenting the mean (*n* = 3) ± Standard deviation. Various lowercase letter superscripts indicate statistical significance at 95% confidence interval. IR indicates irrigation regimes, culti. represents cultivars, and AAB indicates activated acacia biochar.

**Table 3:** Means comparison for the effect of varying irrigation regimes, AAB and cultivars on root dry weight (g), plant height (cm), root length (cm), number of leaves, and number of tillers of wheat.

		**Root dry weight (g)**			**Plant height (cm)**			**Root length (cm)**			**Number of leaves**			**Number of tillers**		
**Culti.**	**IR**	**0T-AAB**	**5T-AAB**	**10T-AAB**	**0T-AAB**	**5T-AAB**	**10T-AAB**	**0T-AAB**	**0T-AAB**	**0T-AAB**	**0T-AAB**	**5T-AAB**	**10T-AAB**	**0T-AAB**	**5T-AAB**	**10T-AAB**
**Dilkash-2020**	**100%IR**	1.75 ± 0.12^cd^	2.42 ± 0.04^bc^	2.44 ± 0.1^bc^	114.13 ± 0.49^e^	118.13 ± 1.33^b^	121.36 ± 2.23^a^	15.80 ± 0.80^b^	14.30 ± 0.20^bc^	15.30 ± 0.20^b^	27.66 ± 1.5^c^	29.33 ± 2.51^b^	32.66 ± 3.51^a^	2.66 ± 1.15^d^	3.66 ± 0.35^c^	6.00 ± 1.00^a^
	**80%IR**	1.24 ± 0.12^fg^	2.23 ± 0.03^bc^	2.30 ± 0.2^bc^	112.80 ± 0.40^ef^	115.13 ± 1.59^d^	118.26 ± 0.50^b^	14.60 ± 0.10^bc^	13.53 ± 0.40^c^	14.53 ± 0.40^bc^	23.33 ± 0.5^cd^	25.00 ± 0.00^bc^	28.66 ± 1.5^b^	2.66 ± 0.57^d^	3.66 ± 0.5^c^	5.66 ± 0.57^ef^
	**70%IR**	1.04 ± 0.04^fgh^	1.90 ± 0.10^cd^	1.94 ± 0.04^cd^	110.03 ± 1.65^fg^	111.56 ± 1.51^ef^	117.30 ± 0.45^c^	12.93 ± 0.23^bc^	11.63 ± 0.86^bcd^	12.63 ± 0.86^bcd^	20.33 ± 1.5^de^	23.66 ± 1.5^cd^	25.00 ± 0.0^bc^	4.00 ± 1.00^bc^	3.00 ± 0.00^c^	4.33 ± 0.57^bc^
	**60%IR**	0.70 ± 0.16^gh^	1.49 ± 0.20^ef^	1.62 ± 0.12^de^	104.40 ± 3.61^fgh^	108.46 ± 1.24^efg^	114.50 ± 0.79 ^e^	10.26 ± 1.06^d^	10.50 ± 0.10^d^	11.50 ± 1.00^cd^	17.33 ± 1.5^efg^	19.00 ± 1.00^def^	22.00 ± 2.6^cd^	4.33 ± 0.57^bc^	2.66 ± 0.57^d^	4.66 ± 0.57^bc^
	**50% IR**	0.46 ± 0.05^ghi^	1.24 ± 0.02^fg^	1.41 ± 0.02^ef^	95.60 ± 2.25^ghi^	103.96 ± 2.30^gh^	111.33 ± 3.52^ef^	6.50 ± 1.60^gh^	9.00 ± 0.78^de^	10.00 ± 0.78^d^	13.00 ± 0.00^ghi^	15.33 ± 1.5^gh^	16.66 ± 1.5^fg^	3.66 ± 0.57^c^	3.00 ± 0.00^c^	4.33 ± 0.57^bc^
**Akbar-2019**	**100%IR**	2.71 ± 0.25^ef^	2.63 ± 0.20^ef^	3.10 ± 0.12^a^	108.76 ± 3.51^efg^	110.10 ± 3.55^fg^	112.50 ± 4.1^ef^	19.40 ± 2.02^a^	14.16 ± 0.98^b^	15.70 ± 1.03^b^	22.66 ± 1.15^cd^	25.33 ± 1.15^bc^	28.66 ± 2.1^b^	5.33 ± 0.57^ef^	3.33 ± 1.15^c^	3.00 ± 0.00^c^
	**80%IR**	2.08 ± 0.02^cd^	2.16 ± 0.25^c^	2.76 ± 0.17^ef^	102.80 ± 0.70^fgh^	103.60 ± 0.96^gh^	105.73 ± 0.41^gh^	14.06 ± 0.72^bc^	11.73 ± 0.47^bcd^	13.50 ± 0.87^bc^	18.66 ± 1.15^ef^	20.66 ± 1.15^de^	21.66 ± 1.2^cde^	3.00 ± 1.00^bc^	4.33 ± 1.52^bc^	5.00 ± 0.00^ef^
	**70%IR**	1.73 ± 0.22^cd^	1.49 ± 0.06^ef^	2.25 ± 0.34^bc^	100.86 ± 0.75^gh^	101.50 ± 0.45^gh^	104.86 ± 0.15^gh^	10.93 ± 0.72^d^	10.36 ± 0.92^d^	12.00 ± 0.20^bcd^	17.00 ± 1.00^efg^	18.33 ± 1.15^ef^	20.66 ± 0.6^de^	2.66 ± 0.56^cd^	3.00 ± 0.00^c^	4.00 ± 0.00^bc^
	**60%IR**	1.22 ± 0.04^fg^	1.08 ± 0.07^fgh^	1.70 ± 0.18^cd^	98.23 ± 0.86^ghi^	100.30 ± 0.45^gh^	103.96 ± 0.25^gh^	9.03 ± 1.41^de^	8.70 ± 0.10^ef^	9.70 ± 0.10^de^	13.00 ± 1.7^ghi^	16.66 ± 0.00^fg^	18.33 ± 0.56^ef^	3.33 ± 0.57^c^	3.00 ± 1.00^c^	3.33 ± 0.5^c^
	**50% IR**	0.65 ± 0.04^gh^	0.61 ± 0.07^gh^	1.50 ± 0.02 ^cdf^	92.20 ± 4.2^ghi^	94.76 ± 4.47^ghi^	100.43 ± 3.42^gh^	6.30 ± 0.66^gh^	7.16 ± 0.94^fg^	7.56 ± 0.80 ^egf^	12.00 ± 0.00 ^i^	14.33 ± 2.08^gh^	14.00 ± 2.0^gh^	3.33 ± 0.57^c^	4.00 ± 1.00^bc^	3.66 ± 1.15^c^
**FSD-08**	**100%IR**	3.66 ± 1.00^a^	2.62 ± 0.24^ef^	2.65 ± 0.22^a^	109.30 ± 2.21^fg^	111.00 ± 2.45^ef^	112.93 ± 1.04^ef^	17.93 ± 0.40^ef^	13.96 ± 0.45^c^	15.03 ± 0.35^ef^	25.66 ± 3.7^bc^	27.66 ± 4.7^c^	30.00 ± 4.5^ef^	3.33 ± 0.5^c^	3.33 ± 1.15^c^	4.33 ± 1.15^bc^
	**80%IR**	2.87 ± 0.07^ef^	1.73 ± 0.17^cd^	2.30 ± 0.22^ef^	102.53 ± 1.76^fgh^	104.10 ± 2.13^gh^	108.20 ± 3.21^efg^	13.26 ± 1.58^c^	12.43 ± 0.41^bcd^	13.83 ± 0.70^c^	22.33 ± 0.5^cd^	24.00 ± 0.00^bc^	22.66 ± 2.5^cd^	3.33 ± 1.5^c^	3.66 ± 0.5^c^	4.33 ± 0.5^bc^
	**70%IR**	0.91 ± 0.07^fgh^	1.33 ± 0.13^efg^	1.96 ± 0.05^cd^	95.33 ± 2.30^ghi^	101.46 ± 0.94^fgh^	102.30 ± 0.79^gh^	10.93 ± 0.70^d^	11.10 ± 0.20^cd^	12.10 ± 0.20^bcd^	19.33 ± 1.5 ^e^	20.88 ± 1.5^de^	20.33 ± 0.6^de^	4.66 ± 0.5^bc^	4.33 ± 0.5^bc^	4.66 ± 0.5^bc^
	**60%IR**	0.58 ± 0.06^ghi^	0.99 ± 0.06^gh^	1.77 ± 0.13^d^	92.13 ± 0.86^ghi^	96.50 ± 0.43^ghi^	98.67 ± 0.70^ghi^	8.60 ± 1.50^ef^	8.90 ± 0.70^ef^	9.80 ± 0.56^de^	16.33 ± 1.5^fg^	17.33 ± 2.1^efg^	18.33 ± 1.5^ef^	4.00 ± 0.00^b^	3.33 ± 0.5^c^	3.66 ± 0.5^c^
	**50% IR**	0.28 ± 0.04 ^i^	0.73 ± 0.17^gh^	1.57 ± 0.03^ef^	89.46 ± 0.90^hi^	94.70 ± 0.43^ghi^	95.26 ± 1.00^ghi^	6.30 ± 0.50^gh^	6.76 ± 0.89 ^gf^	8.30 ± 0.95^ef^	12.00 ± 2.6 ^i^	13.00 ± 2.0^ghi^	14.66 ± 1.5^gh^	3.66 ± 1.15^c^	4.00 ± 1.00^bc^	3.66 ± 0.5^c^

**Notes.**

Data presenting the mean (*n* = 3) ± Standard deviation. Various lowercase letter superscripts indicate statistical significance at 95% confidence interval. IR indicates irrigation regimes, culti. represents cultivars, and AAB indicates activated acacia biochar.

**Table 4:** Means comparison for the effect of varying irrigation regimes, AAB and cultivars on spike length (cm), spike weight (g), number of spikes/plant, number of grains/spike, number of spikelet/spikes, 1000-grain weight (g) of wheat.

		**Spike length (cm)**			**Spike weight (g)**			**Number of spikes/plants**			**Number of grains/spikes**			**Number of spikelet/spikes**			**1000-Grain weight (g)**		
**Cult.**	**IR**	**0T-AAB**	**5T-AAB**	**10T-AAB**	**0T-AAB**	**5T-AAB**	**10T-AAB**	**0T-AAB**	**5T-AAB**	**10T-AAB**	**0T-AAB**	**5T-AAB**	**10T-AAB**	**0T-AAB**	**5T-AAB**	**10T-AAB**	**0T-AAB**	**5T-AAB**	**10T-AAB**
**Dilkash-2020**	**100%IR**	13.5 ± 1.3^cd^	14.5 ± 1.32^bc^	19.2 ± 1.36^a^	12.6 ± 2^b^	14.8 ± 2.9^ef^	16.8 ± 2.2^a^	5.0 ± 0.0^b^	6.0 ± 0.00^ef^	6.3 ± 0.5^a^	68.7 ± 1.5^b^	59.3 ± 1.84^d^	70.3 ± 1.5^ef^	21.6 ± 0.5^bc^	21.6 ± 0.5^bc^	23.0 ± 0.0^ef^	54.4 ± 0.9^bc^	56.4 ± 0.8^b^	61.5 ± 1.2^a^
	**80%IR**	12.1 ± 0.8^cd^	13.1 ± 0.8^cd^	17.7 ± 0.30^b^	8.7 ± 0.3^ef^	10.7 ± 0.9^cd^	11.1 ± 0.82^bc^	3.3 ± 0.5^c^	5.0 ± 0.00^b^	3.7 ± 0.5^bc^	64.0 ± 1.0^cd^	56.3 ± 1.9^d^	63.7 ± 1.5^cd^	20.0 ± 0.0^bcd^	19.3 ± 1.7^d^	19.0 ± 0.0^d^	49.7 ± 1.4^de^	50.6 ± 0.4^d^	55.9 ± 1.9^bc^
	**70%IR**	15.1 ± 1.8 ^abc^	16.2 ± 1.8^ef^	17.4 ± 1.5^b^	7.7 ± 0.1^fg^	8.7 ± 0.5^ef^	9.3 ± 0.2^de^	2.0 ± 0.0^d^	4.3 ± 0.5^bc^	5.0 ± 0.00^b^	60.3 ± 0.5^cde^	72.6 ± 1.1^a^	68.3 ± 0.5^b^	19.0 ± 1.0^d^	21.0 ± 0.0^bc^	24.3 ± 0.5^a^	45.4 ± 0.9^fg^	51.9 ± 0.8^cd^	56.3 ± 0.1^b^
	**60%IR**	11.2 ± 1.1^de^	12.2 ± 1.1^cd^	14.0 ± 0.5^bc^	7.1 ± 0.1^fg^	7.7 ± 0.18^fg^	8.6 ± 0.5^ef^	2.0 ± 0.0^d^	3.3 ± 0.5^c^	3.3 ± 0.5^c^	58.3 ± 0.5^d^	67.0 ± 3.0^c^	62.7 ± 0.5^cd^	16.6 ± 1.1^efg^	18.0 ± 0.0^de^	17.0 ± 0.0^ef^	42.5 ± 0.4^gh^	49.6 ± 0.2^de^	50.3 ± 0.9^d^
	**50% IR**	11.7 ± 1.1^de^	12.8 ± 1.1^cd^	13.6 ± 0.3^cd^	5.6 ± 1.2^hi^	5.9 ± 0.1^hi^	6.4 ± 1.2^gh^	2.0 ± 0.0^d^	2.3 ± 0.5^cd^	3.0 ± 0.5^c^	56.0 ± 1.0^de^	59.7 ± 0.5^d^	60.3 ± 0.5^cde^	16.3 ± 0.5^efg^	16.6 ± 1.1^efg^	19.6 ± 1.5^d^	39.9 ± 0.5^ghi^	47.3 ± 0.4^def^	47.9 ± 0.6^def^
**Akbar-2019**	**100%IR**	11.5 ± 1.4^de^	12.5 ± 1.4^cd^	14.2 ± 2.2^bc^	10.3 ± 1.4^cd^	11.1 ± 1.5^bc^	11.3 ± 2.3^bc^	3.7 ± 1.5^bc^	5.0 ± 0.00^b^	4.3 ± 0.5^bc^	65.0 ± 1.0^bc^	57.0 ± 1.0^d^	65.7 ± 0.5^bc^	19.0 ± 0.0^d^	20.3 ± 1.1^bcd^	19.7 ± 3.2^d^	52.7 ± 0.5^bcd^	51.1 ± 1.3^cd^	53.2 ± 1.0^bc^
	**80%IR**	11.7 ± 0.7^de^	12.7 ± 0.7^cd^	10.9 ± 0.5^ef^	6.6 ± 0.2^gh^	7.8 ± 0.6^fg^	8.0 ± 0.6^ef^	3.3 ± 0.5^c^	3.7 ± 0.5^bc^	3.0 ± 0.00^c^	62.8 ± 0.5^c^	66.7 ± 1.5^bc^	60.7 ± 0.5^cde^	16.7 ± 0.5^efg^	18.0 ± 0.0^de^	18.3 ± 3.2d ^e^	48.7 ± 1.3^de^	48.7 ± 1.3^de^	48.6 ± 0.6^def^
	**70%IR**	10.5 ± 0.1^ef^	11.5 ± 0.8^de^	11.4 ± 0.2^de^	5.5 ± 0.2^hi^	6.2 ± 0.2^gh^	6.6 ± 0.2^gh^	2.3 ± 0.5^cd^	4.0 ± 0.0^bc^	4.0 ± 0.00^bc^	59.7 ± 1.5^de^	62.0 ± 1.0^cd^	61.7 ± 0.5^cde^	15.7 ± 0.5^fg^	21.0 ± 0.0^bc^	19.6 ± 4.0^d^	41.4 ± 1.0^gh^	50.4 ± 0.2^d^	45.7 ± 1.3^fg^
	**60%IR**	10.3 ± 0.1^ef^	11.3 ± 0.1^de^	11.7 ± 0.6^de^	5.1 ± 0.1^hi^	5.3 ± 0.1^hi^	6.1 ± 0.2^gh^	2.0 ± 0.0^d^	3.0 ± 0.0c	3.0 ± 0.00^c^	55.7 ± 1.5^de^	58.0 ± 1.7^de^	57.7 ± 1.5^de^	14.3 ± 0.5^gh^	17.0 ± 0.0^ef^	15.3 ± 0.5^fg^	39.3 ± 0.5^ghi^	47.0 ± 1.7^def^	44.4 ± 0.8^fg^
	**50% IR**	11.2 ± 1.4^de^	10.1 ± 0.7^ef^	10.8 ± 0.8^ef^	3.1 ± 0.9^j^	4.3 ± 0.1^ij^	4.5 ± 0.85^ij^	1.3 ± 0.5^de^	2.3 ± 0.5^cd^	3.0 ± 1.00^c^	52.7 ± 0.5^efg^	53.7 ± 0.5^ef^	53.3 ± 1.5^ef^	13.0 ± 0.0^gh^	16.0 ± 0.0^efg^	15.6 ± 1.1^fg^	37.1 ± 0.9^hi^	44.1 ± 0.6^fg^	43.5 ± 0.5^fgh^
**FSD-08**	**100%IR**	11.6 ± 1.3^de^	12.6 ± 1.3^cd^	10.2 ± 0.8^ef^	8.7 ± 1.5^ef^	9.1 ± 1.1^de^	11.3 ± 2.3^bc^	4.3 ± 0.5^bc^	4.0 ± 0.0^bc^	4.0 ± 1.00^bc^	64.0 ± 2.0^c^	63.7 ± 2.0^cd^	63.0 ± 2.0^cd^	20.0 ± 0.0^bcd^	20.3 ± 0.5^bc^	18.0 ± 0.0^de^	52.0 ± 1.6^bcd^	52.9 ± 0.6^bcd^	50.7 ± 0.5^d^
	**80%IR**	9.9 ± 0.7^fg^	10.9 ± 0.7^ef^	13.4 ± 3.1^bc^	6.3 ± 0.3^gh^	6.8 ± 0.7^gh^	7.3 ± 0.6^fg^	4.0 ± 0.0^bc^	3.0 ± 0.0^cd^	3.3 ± 0.5^c^	61.3 ± 1.5^cd^	53.0 ± 1.0^ef^	55.7 ± 2.0^de^	18.0 ± 0.0^de^	18.6 ± 0.5^de^	15.6 ± 1.5^fg^	46.7 ± 3.6^efg^	49.2 ± 1.1^de^	47.3 ± 0.2^ef^
	**70%IR**	9.6 ± 0.6^fg^	10.6 ± 0.6^ef^	11.4 ± 0.2^de^	5.2 ± 0.4^hi^	5.7 ± 0.2^hi^	6.11 ± 0.2^gh^	3.0 ± 0.0^c^	4.0 ± 0.0^bc^	4.3 ± 0.5^bc^	57.0 ± 1.0^de^	57.7 ± 4.0^de^	61.0 ± 1.0^cde^	16.0 ± 0.0^efg^	22.0 ± 0.0^ef^	20.0 ± 0.0^bc^	42.5 ± 1.1^gh^	50.4 ± 0.8^d^	44.9 ± 0.5^fgh^
	**60%IR**	9.8 ± 0.6^fg^	10.9 ± 0.6^ef^	10.8 ± 0.8^ef^	4.3 ± 0.1^ij^	4.5 ± 0.1^ij^	5.6 ± 0.23^hi^	3.0 ± 0.0^c^	2.3 ± 0.5^cd^	2.7 ± 0.5^cd^	52.3 ± 0.5^efg^	55.6 ± 2.8^de^	53.0 ± 1.0^ef^	14.0 ± 0.0^gh^	17.3 ± 0.5^ef^	16.0 ± 0.0^efg^	39.3 ± 0.8^ghi^	45.6 ± 0.9^fg^	43.4 ± 0.5^fgh^
	**50% IR**	9.1 ± 1.3^g^	10.2 ± 0.7^ef^	9.6 ± 0.7^efg^	3.4 ± 0.5^j^	4.2 ± 0.1^ij^	4.5 ± 0.8^ij^	2.0 ± 0.0^d^	2.7 ± 0.5^cd^	2.7 ± 0.5^cd^	51.0 ± 1.0^fg^	52.0 ± 1.0^rfg^	52.0 ± 1.0^rfg^	12.7 ± 0.5^i^	16.7 ± 0.5^efg^	15.0 ± 0.0^fg^	35.0 ± 3.1^i^	41.1 ± 0.6^ghi^	42.1 ± 0.6^gh^

**Notes.**

Data presenting the mean (*n* = 3) ± standard deviation. Various lowercase letter superscripts indicate statistical significance at 95% confidence interval. IR indicates irrigation regimes, culti. represents cultivars, and AAB indicates activated acacia biochar.

### Pearson correlation

**Figure 6:**
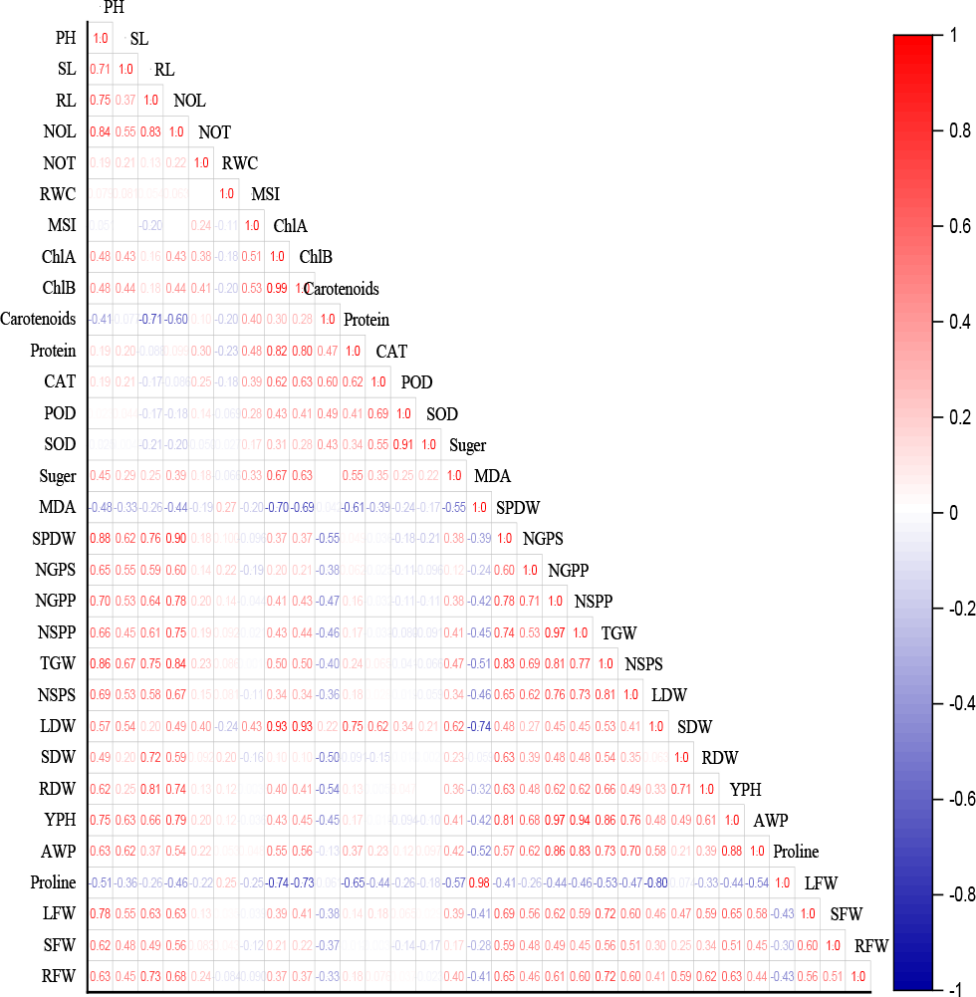
Pearson correlation for interaction among observed traits for all cultivars of wheat under deficit irrigation with AB amendments under varying IR levels (* significance at *p* ≤0.05). PH, plant height; SL, spike length; RL, root length; NOL, number of leaves; NOT, number of tillers; RWC, relative water content; MSI, membrane stability index; ChlA, chlorophyll a; ChlB, chlorophyll b; CAT, catalase; POD, peroxidase; SOD, superoxide dismutase; MDA, malondialdehyde; SPDW, spike dry weight; NGPS, number of grain per spike; NGPP, number of grain per plant; NSPP, number of spikes per plant; TGW, thousand grain weight, number of spikelet per spike, LDW, leaf dry weight; SDW, stem dry weight; RDW, root dry weight; YP, yield per hectare; AWP, apparent water productivity; LFW, leaf fresh weight; SFW, stem fresh weight; RFW, root fresh weight.

Pearson correlation among all the observed traits was performed to understand their correlation with the most relevant traits (Fig. 6). Wheat morphological attributes as well as yield attributes were positively correlated. As the red color in the plot presents a positive association between two traits and blue shows a negative correlation, whereas the white color shows no correlation among traits. It was observed that carotenoids, MDA, proline, POD, and SOD significantly had a negative correlation with all other traits.

### Multivariate analysis

**Figure 7:**
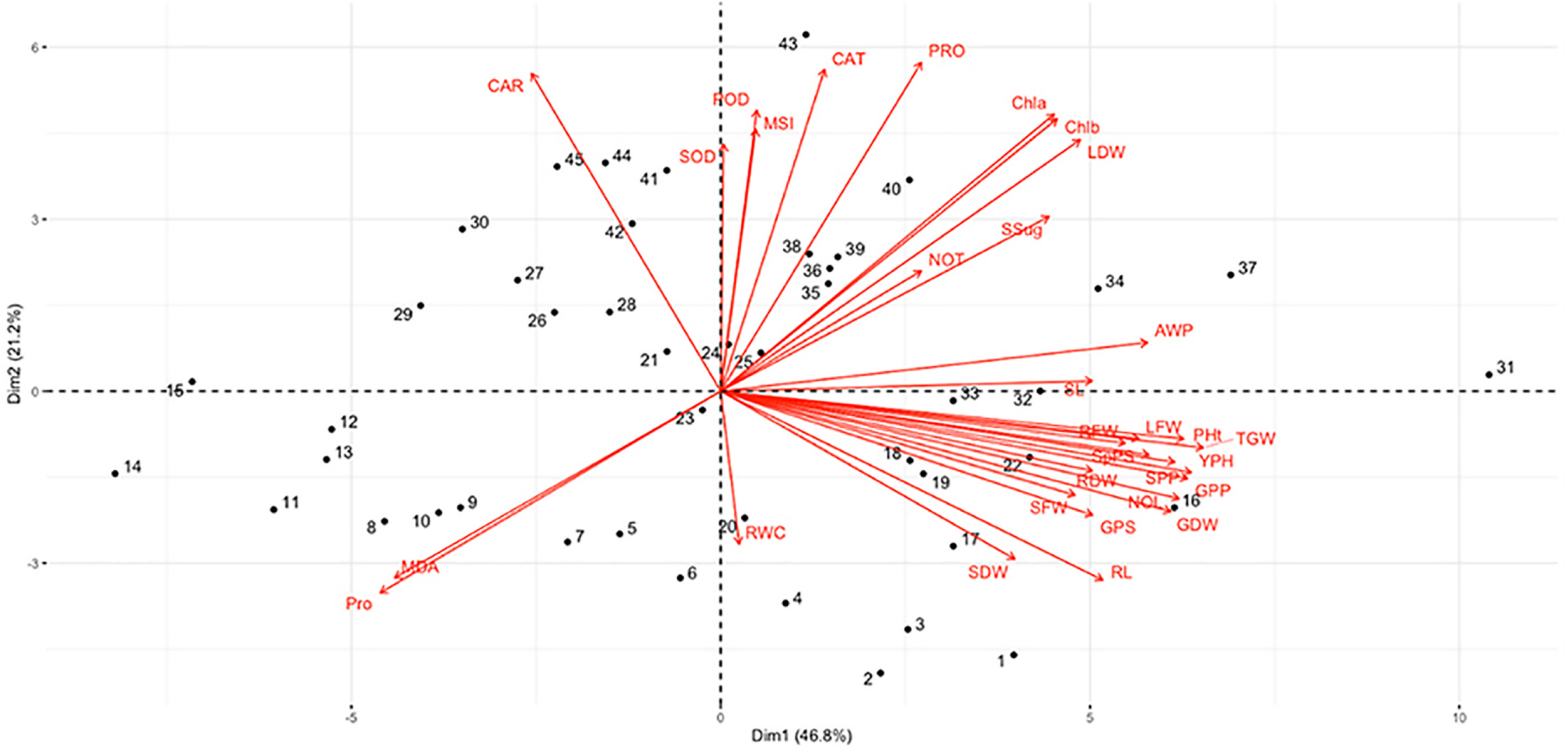
Principal component analysis (PCA) Biplot showing the effect of varying irrigation regimes, AB and cultivars on plant responses. PH, plant height; SL, spike length; RL, root length; NOL, number of leaves; NOT, number of tillers; RWC, relative water content; MSI, membrane stability index; ChlA, chlorophyll a; ChlB, chlorophyll b; CAT, catalase; POD, peroxidase; SOD, superoxide dismutase; MDA, malondialdehyde; PRO, protein; Pro, proline; SPDW, spike dry weight; NGPS, number of grain per spike; NGPP, number of grain per plant; NSPP, number of spikes per plant; TGW, thousand grain weight, number of spikelet per spike; LDW, leaf dry weight; SDW, stem dry weight; RDW, root dry weight; YP, yield per hectare; AWP, apparent water productivity; LFW, leaf fresh weight; SFW, stem fresh weight; RFW, root fresh weight. **1** = 0T AAB+ 100% IR+ Dilkash, **2** = 0T AAB+ 80% IR+ Dilkash, **3** = 0T AAB+ 70% IR+ Dilkash, **4** = 0T AAB+ 60% IR+ Dilkash, **5** = 0T AAB+ 50% IR+ Dilkash, **6** = 0T AAB+ 100% IR+ Akbar, **7** = 0T AAB+ 800% IR+ Akbar, **8** = 0T AAB+ 70% IR+ Akbar, **9** = 0T AAB+ 600% IR+ Akbar, **10** = 0T AAB+ 500% IR+ Akbar, **11** = 0T AAB+ 100% IR+ FSD-08, **12** = 0T AAB+ 80% IR+ FSD-08, **13** = 0T AAB+ 70% IR+ FSD-08, **14** = 0T AAB+ 60% IR+ FSD-08, **15** = 0T AAB+ 50% IR+ FSD-08, **16** = 5T AAB+ 100% IR+ Dilkash, **17** = 5T AAB+ 80% IR+ Dilkash, **18** = 5T AAB+ 70% IR+ Dilkash, **19** = 5T AAB+ 60% IR+ Dilkash, **20** = 5T AAB+ 50% IR+ Dilkash, **21** = 5T AAB+ 100% IR+ Akbar, **22** = 5T AAB+ 800% IR+ Akbar, **23** = 5T AAB+ 70% IR+ Akbar, **24** = 5T AAB+ 600% IR+ Akbar, **25** = 5T AAB+ 500% IR+ Akbar, **26** = 5T AAB+ 100% IR+ FSD-08, **27** = 5T AAB+ 80% IR+ FSD-08, **28** = 5T AAB+ 70% IR+ FSD-08, **29** = 5T AAB+ 60% IR+ FSD-08, **30** = 5T AAB+ 50% IR+ FSD-08, **31** = 10T AAB+ 100% IR+ Dilkash,** 32** = 10T AAB+ 80% IR+ Dilkash, **33** = 10T AAB+ 70% IR+ Dilkash, **34** = 10T AAB+ 60% IR+ Dilkash, **35** = 10T AAB+ 50% IR+ Dilkash, **36** = 10T AAB+ 100% IR+ Akbar, **37** = 10T AAB+ 800% IR+ Akbar, **38** = 10T AAB+ 70% IR+ Akbar, **39** = 10T AAB+ 600% IR+ Akbar, **40** = 10T AAB+ 500% IR+ Akbar, **41** = 10T AAB+ 100% IR+ FSD-08, **42** = 10T AAB+ 80% IR+ FSD-08, **43** = 10T AAB+ 70% IR+ FSD-08, **44** = 10T AAB+ 60% IR+ FSD-08, **45** = 10T AAB+ 50% IR+ FSD-08, where Dilkash = Dilkash-2020 cultivar, Akbar = Akbar-2019 cultivar, and FSD-08 = FSD-O8 cultivar.

**Figure 8:**
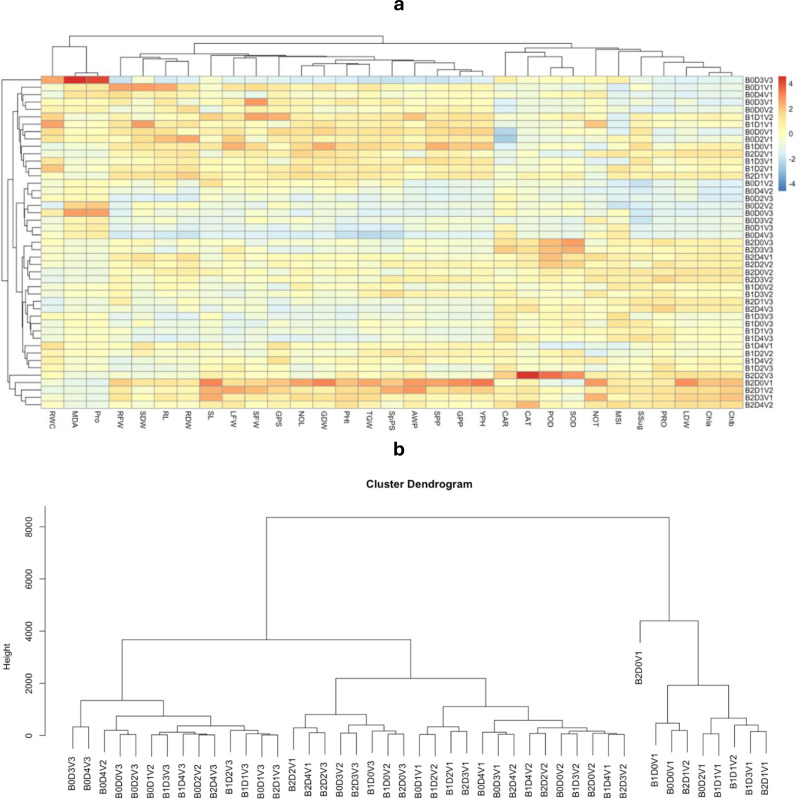
Heatmap showing effective significance of AAB under varying irrigation regimes on wheat cultivars B0 = 0T-AAB, B1 = 5T-AAB, B2 = 10T-AAB, D0 = 100% IR, D1 = 80% IR, D2 = 70% IR, D3 = 60% IR, D4 = 50% IR, V1 = Dilkash-2020, V2 = Akbar-2019, and V3 = FSD-08.

This study utilizes principal component analysis (PCA) heatmap and biplot to explore the relationship between activated acacia biochar amended soil under varying irrigation regimes cultivated with different wheat cultivars and physiological, biochemical, and yield variables (Fig. 7). The analysis effectively distinguished plants exposed to deficit irrigation with activated biochar amended and non-amended soil. The heatmap clearly showed that the changes made by 10T-AAB had the most significant impacts on morphological, physiological, and yield attributes (Fig. 8A). The most prominent effect among morphological traits with 10T-AAB level of soil amendment were observed in number of leaves, plant height, and plant fresh and dry weights. Whereas among physiological traits, a significant effect of activated biochar amendment was observed for plant relative water content, membrane stability index, chlorophyll contents, and antioxidants under deficit irrigations. However, 0T-AAB had the highest levels of proline content, catalase, peroxidase, and superoxide dismutase, indicating the severity of stress. The biplot presented two main clusters each representing a group of applied treatments (Fig. 8B).

## Discussion

Among several types of biochar, the one produced using wood biomass exhibits a large surface area due to the higher lignin content of the wood. Further, its activation with organic wastes adds valuable and promising properties (Jahan et al., 2023). The current study showed that increased water holding capacity with activated biochar application is consistent with ameliorating soil health and fertility. Biochar improved soil water retention by improving the soil’s micropore structure and reducing the macropore surface (Abel et al., 2013). This reduction was attributed to biochar’s ability to fill macropore surfaces in soil, influencing pore size distribution and leading to higher soil density with increased micropore proportion. Moreover, biochar itself has a porous nature, which adds to the total soil pore volume, improving aerations and water infiltration (Karhu et al., 2011). Under biochar amendment, soil pH was increased from acidic to neutral, which can be a consequence of the liming effect of biochar due to the presence of basic cations such as magnesium, calcium, and potassium (Yuan & Xu, 2011). Variation in electrical conduction of soil with AAB amendment showed that biochar initially releases soluble salts, thereby increasing the electrical conductivity of soil, but over time, these salts are utilized by plants or leached away (Major et al., 2010). Moreover, carbon recovery, mean residence time, and carbon sequestration are a consequence of recalcitrant carbon components in acacia activated biochar that remain in soil over a longer period.

The levels of proline and lipid peroxidation increased in water deficit conditions. But biochar reduced these stress markers by improving soil water retention in current study which is in agreement with the findings of Gharred et al. (2022). Plants usually accelerate their antioxidant activity to cope with reactive oxygen species (ROS) produced under abiotic stress (Mu et al., 2021). The current study observed an increased level of protein content but reduced antioxidants (CAT, POD, and SOD) with biochar application under low irrigation regimes, especially 60% and 50%. This effect was attributed to biochar’s ability to enhance ROS scavenging mechanisms, reducing oxidative stress (Nawaz et al., 2023), which in turn provides protection against lipid damage (Farhangi-Abriz & Torabian, 2017), as can be evidenced by reduced proline, MDA contents, and antioxidant levels.

Sugar content, RWC, and MSI were enhanced with 10T-AAB even under low irrigation levels. Improved sugar contents were associated with reduced osmotic stress and enhanced soil fertility due to increased soil organic carbon (SOC). Higher SOC levels with biochar amendment lead to improved nutrient availability and metabolic activities (Jahan et al., 2023). Tanure et al. (2019) stated that biochar’s porous structure enhances water retention that helps maintain higher RWC in plant tissues, thereby improving cellular metabolism. Drought stress directly affects the photosynthetic ability of plants, which can be ameliorated using biochar (Sattar et al., 2019). Photosynthetic pigments were observed to be increased with increasing levels of AAB and played a role in mitigating the negative effects of a low irrigation regime. These increased levels with biochar were attributed to enhanced nutrients particularly nitrogen, which is crucial component of porphyrin ring of chlorophyll molecule, which in turn promoted chlorophyll biosynthesis (Abideen et al., 2020). Additionally, the study observes that biochar alters soil pH, influencing nutrient absorption and availability in the rhizosphere (Ayaz et al., 2021). The observed improved soil structure contributes to better plant development (Manolikaki & Diamadopoulos, 2019).

Plants treated with activated biochar showed enhanced root growth, which can be attributed to improved soil structure and increased nutrient availability (Zhao et al., 2019). According to Jahan et al. (2023), biochar is characterized by high surface area enhancing soil water holding capacity, which improves soil aeration and reduces soil compaction, allowing extensive root growth. As previously indicated, biochar can improve root growth in plants, which is essential for increasing water use efficiency and plant survival during dry spells (Zulfiqar et al., 2022; Tanure et al., 2019).

The present results demonstrated that biochar amendments contribute to reducing losses in wheat growth and yield by retaining water in soil pores and gradually releasing it under moisture deficit, like the findings of Ali et al. (2017). Activated biochar positively influenced spike development, which can be attributed to enhanced chlorophyll contents, relative water contents, protein accumulation, and increased soil nutrients especially carbon and nitrogen, essential for plant reproductive growth.

The number of spikelets per spike along with grain filling was higher in 10T-AAB treated plants leading to heavier grains as compared to control, which is according to the findings of Haider et al. (2020), indicating better grain quality and yield with biochar application even under low irrigation regimes (Zulfiqar et al., 2022). Hence, slow crop growth rates, poor source–sink relationships, and malfunctioning metabolic systems contributing to low grain weight under drought stress can be controlled with activated biochar as a soil amendment. However, with activated biochar application, interactive effects of plant growth attributes with soil physicochemical properties and plant’s physiological sustainability against oxidative stress cause resilience to water deficit condition and lead to increased wheat production. Thus, AAB improves stress resilience benefiting wheat growth and yield under water deficit conditions assuring food security.

## Conclusion

The current study illustrates the potential of activated acacia biochar (AB) to enhance wheat crop productivity under deficit water conditions, as can be evidenced by improved apparent water productivity and overall crop yield. The application of AAB resulted in significant improvements in soil physicochemical properties, enhancing soil organic carbon, water holding capacity, electrical conductivity, hydrogen, oxygen, carbon recovery, and carbon sequestration capacity. Furthermore, AAB enhanced drought tolerance in wheat plants by improving biochemical contents, promoting root growth, and enhancing photosynthetic efficiency, as can be witnessed by enhanced photosynthetic pigments. This soil amendment also enhanced water availability to plants by improving water retention in soil micropores, increasing crop resilience to deficit irrigations. These positive effects ultimately translated into improved yield attributes, such as increased grain yield. Overall, the findings highlight the promising role of AAB as a sustainable agricultural practice for mitigating the adverse impacts of water scarcity on wheat cultivation. Further investigations are necessary to optimize application rates and assess long-term effects on soil-plant-water interactions and crop productivity. As activated acacia biochar plays a role in remediation of degraded soil health, hence, soil microbiome under the effect of biochar can be studied further. Moreover, there is need to integrate activated biochar in agricultural soils to combat deficit water conditions because of climate change which needs technical training of people associated with agricultural sectors especially farmers about the impact of activated biochar.

## Supplemental Information

10.7717/peerj.18748/supp-1 Supplemental Information 1 R Codes for PCA analysis and Heatmap analysis

10.7717/peerj.18748/supp-2 Supplemental Information 2 Physiological, biochemical and yield attributes of wheat under varying irrigation regimes

## Additional Information and Declarations

### Data Availability

The following information was supplied regarding data availability: The codes used for multivariate analysis and raw measurements are available in the Supplementary Files.
